# Relative Food Prices and Obesity in U.S. Metropolitan Areas: 1976-2001

**DOI:** 10.1371/journal.pone.0114707

**Published:** 2014-12-12

**Authors:** Xin Xu, Jayachandran N. Variyam, Zhenxiang Zhao, Frank J. Chaloupka

**Affiliations:** 1 Institute for Health Research and Policy, University of Illinois at Chicago, Chicago, Illinois, United States of America; 2 Food Economics Division, Economic Research Service, U. S. Department of Agriculture, Washington, District of Columbia, United States of America; 3 Institute for Health Research and Policy, Department of Economics, University of Illinois at Chicago, National Bureau of Economic Research, Chicago, Illinois, United States of America; CUNY, United States of America

## Abstract

This study investigates the impact of food price on obesity, by exploring the co-occurrence of obesity growth with relative food price reduction between 1976 and 2001. Analyses control for female labor participation and metropolitan outlet densities that might affect body weight. Both the first-difference and fixed effects approaches provide consistent evidence suggesting that relative food prices have substantial impacts on obesity and such impacts were more pronounced among the low-educated. These findings imply that relative food price reductions during the time period could plausibly explain about 18% of the increase in obesity among the U.S. adults in metropolitan areas.

## Introduction

Body weight has risen dramatically in the United States since early 1970s and most of this increase occurred in the 1980s and 1990s [1]–[4]. The prevalence of obesity in the U.S. was 16.9% in youth and 34.9% in adults in 2010–2011 [4].

The estimated annual cost of treating obesity in the U.S. adult non- institutionalized population was $168.4 billion, 16.5% of national spending on medical care [5]. Without any effective policy interventions, the medical costs associated with obesity would rise by $46–$88 billion annually by 2030 [6]. Roughly half of these medical costs have been financed by Medicare or Medicaid creating a significant externality on other members of the society [7]. Even in private-group health insurance programs, the negative externality resulting from medical bills associated with diseases and treatments related to obesity may also raise the insurance premiums for all members enrolled in the programs [8].

A wide range of public policies to curb the epidemic of obesity have been discussed, although the complex range of social and economic factors that drive the energy imbalance and lead to the epidemic is not fully understood. However, both economic and public health literature generally agree that dietary overconsumption related to technological innovations is one of the major reasons for this epidemic [1], [8]–[18]. In particular, one recent study has shown that energy intake is mainly responsible for the sharp increase in body weight in developed countries and that the three leading social factors of energy intake are the relative food price, female labor force participation, and urbanization defined as percentage population living in urban areas [16]. Among these three factors, the relative food price, which reflects changes in cost of food relative to all consumer goods, is the one that could be effectively and legitimately affected by public policies.

Previous literature related to food prices and obesity can be characterized into two groups: studies examining the association between food prices and food consumption and studies exploring the association between food prices and body weight. The former set of studies usually focuses on the price elasticity of one or several food items [19]–[30]. The latter takes a reduced form approach to evaluate the effect of food price on body weight and most of them focus on the impacts of absolute prices [8], [11], [12], [27], [31]–[44] Moreover, most of these studies are based on multiple cross-sectional data, except some recent studies [37], [38], [42], [44], [45]. A few studies have investigated the impacts of relative food prices in the United States [9], [29], [46]–[48].

The main purpose of this study is to evaluate the effects of relative food prices on the sharp upward obesity trend since 1970s in U.S. large metropolitan areas. The paper makes three contributions to the growing literature on the relationship between food prices and obesity. First, the study takes longitudinal approaches covering the period from 1976 to 2001, nearly the entire duration of the rapid growth in obesity in the U.S. [49]. Second, we used metropolitan consumer price index (CPI) -based food price indexes to capture relative price variation computed from a broad basket of food items. Third, by using CPI-based price indexes for food at home and food away from separately, we disentangle the differential impacts of these prices on obesity. Food away from home is an increasing component of people’s diets and it provides a distinct set of product attributes such as preparation, convenience, and nutrients that may be valued differently compared with food at home attributes.

Specifically, we investigate the effects of relative food prices of food at home and food away from home, using pseudo-panel data constructed from independent cross-sections of the National Health Interview Survey (NHIS) between 1976 and 2001. From both scientific and economic theory perspectives, relative food price is more closely related to food consumption, as individuals maximize their utility by choosing the optimal amount of food under their financial budget constraint, which consists of prices of all other goods in addition to food prices. From an empirical perspective, the relative food price is more plausibly exogenous, as some confounding factors, such as gas price, may simultaneously affect energy intake through food prices and energy expenditure through physical activity. Failing to control for these confounding factors in empirical analysis may lead to biased estimates. By using relative food price, which is the ratio of the food price index to the overall consumer price index, we implicitly control for prices of all goods and services.

In the analysis, we control for the change in female labor force participation during the time period and minimize the influence of urbanization on body weight by focusing on samples living in large metropolitan areas. In addition, we include outlet density measures that may be closely related to body weight, such as the number of grocery stores, restaurants, and fitness and recreational centers to evaluate the potential impact of the omitted variable bias from local specific time-varying factors.

We obtained consistent estimated effects of relative food prices on body weight, by using both the first-difference and fixed effect approaches. Our estimates indicate that the relative prices of food at home and food away from home have substantial impacts on obesity. Such effects are more pronounced for the low-educated. As a result, roughly 18% of the obesity growth between 1976 and 2001 can be explained by the changes in relative food prices.

## Methods

### Empirical Strategy and Specifications

Food price can be an endogenous measure due to unobserved factors that may be correlated with both food prices and other factors associated with obesity. While using relative food prices largely minimizes the endogeneity concern by implicitly controlling for the monetary costs of other commodities, uncontrolled non-monetary factors may still raise the issue.

First-difference and fixed effects (within operators) are procedures commonly used to eliminate biases caused by persistent omitted variables. Both approaches normally require panel data. To the best of our knowledge, no national representative panel data covers the whole time period from 1976 to 2001. To address this issue, we constructed pseudo-panel data using individual NHIS respondents for the analysis [50]. Existing study has shown that independent cross-sections in successive years can be grouped into comparable demographic categories and then differenced to produce many of the advantages gained from differencing individual panel data [50]. For example, grouping into aggregate sample tends to homogenize the individual effects among individuals grouped in the same cell, so that the average specific effect is approximately invariant between two periods and is efficiently removed by within or first-difference transformations.

The concern of pseudo-panel data centers on special errors in measurement when aggregate samples do not contain the same individuals across time periods. This issue can be considered as a measurement error and address it with a Fuller-type correction [50]. However, following studies have shown that Deaton’s pseudo-panel estimator converges with increases in the sample sizes and the number of time periods, because measurement error becomes negligible when samples are large [51], [52]. Consequently, the pseudo-panel data approach has been widely used in a number of studies [53]–[59].

In this analysis, NHIS respondents aged 18 and above are aggregated into cells according to their gender, education, race and ethnicity, geographic location, and interview year. Specifically, we use two categories for gender (male and female), four categories for education (less than high school, high school, some college and college and more), and four categories for race/ethnicity (White, African American, Hispanics, and others). As a result, one aggregate sample (which includes a group of NHIS individual respondents) could be “white, male, high school graduates, living in the New York metropolitan area in year 1976”, and another aggregate sample could be “African American, male, high school graduates, living in New York metropolitan area in year 1976”. The NHIS sampling weights were incorporated when constructing these aggregate samples. Therefore, the aggregate samples in this analysis are both large and homogenous.

We use both first-difference and fixed effect approaches so as to contrast cross-section and panel data estimators for our model. We use the following empirical specifications to define the “between” and “within” transformations:
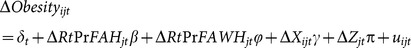
(1)

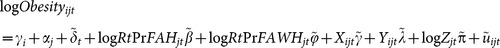
(2)


Equation (1) illustrates the specification of the first-difference approach, where the change in the obesity prevalence of an aggregate sample *i* at metropolitan *j* in year *t* depends on changes in demographic characteristics besides gender, race/ethnicity, and education, such as family income, marital status, and age (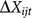), and changes in time-varying factors at metropolitan level (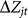), while 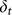 represents year fixed effects. The two parameters of interest are those associated with changes in the relative prices of food at home (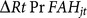) and food away from home (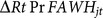). Since dependent variables and key independent variables are measured by changes in percentages, the two parameters of interest (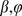) essentially represent price elasticities of demand.

Equation (2) illustrates the specification for the fixed effect approach. The *log* obesity prevalence of an aggregate sample *i* at metropolitan *j* in year *t* is a function of *log* relative price of food at home, *log* relative price of food away from home, metropolitan time-varying factors in *log* unit (*Z_jt_*), and demographic characteristics besides gender, race/ethnicity, and education (*X_ijt_*). Equations (2) also includes additional time-invariant covariates, such as fixed effects of aggregate samples (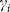, essentially the dummy variable for each group of aggregate samples by gender, race/ethnicity, education, and MSA), indicators of gender, race/ethnicity, and education (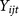), and the metropolitan fixed effects (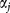).

Therefore, in Equation (1), the data are expressed as deviations from the group means in two consecutive time periods, while in later case the data are of group means in nature log form within the current time period. Note that basing estimation on Equation (2) leads to standard cross-section estimators which may neglect time-varying unobserved heterogeneity across aggregate samples. Thus, if unobserved factors were correlated with changes in relative food prices, such procedures may produce inconsistent estimates. Therefore, this analysis was focused on the first difference approach and the later was mainly used for sensitivity analysis.

Therefore, the major threat to the validity of the causal argument in both approaches is local specific non-price time-varying factors. To address this, metropolitan time-varying factors, such as male wage rates and hours of work per week, unemployment rate and income per capita that may affect body weight are included. In particular, female average wage rates and hours of work are included separately so as to control for the potential effects of female labor participation on body weight [9], [16]. Density measures, including the number of restaurants, grocery or convenience stores, and physical activity facilities per capita are also included, as they may change over time and affect body weight [11], [36], [60]–[69]. More importantly, these density measures may be highly correlated with relative prices of food away from home and food at home, the inclusion/exclusion of these controls in different specifications provides a reasonable test for the causal inference.

Another important aspect of this analysis is the use of pseudo-panel created from individual respondents. One may concern about the sensitivity of our findings to the method used to create the aggregate samples in the pseudo-panel. To address this potential concern, we conducted a sensitivity analysis.

We replicated the analysis using alternative pseudo-panels based on aggregate samples generated from the NHIS individual respondents with different criteria. First, to address the potential concern about the change in educational attainment over the time period, we excluded education as one of the criterion from the aggregating method. Instead, we used only two demographic characteristics, gender and race/ethnicity for the aggregation procedure. In the second scenario, which is an extreme case, we used gender as the only demographic criterion to construct aggregate samples. The underlying rationale is that estimated impacts of relative food prices on body weight should not change substantially if our findings were independent of the aggregation method. As we shown in the following, indeed, that is the case.

Analytical weights generated based on the sample size of each aggregate sample have been included in all analyses to account for the variations in the number of individual respondents within aggregate samples.

### Data

**Figure 1:**
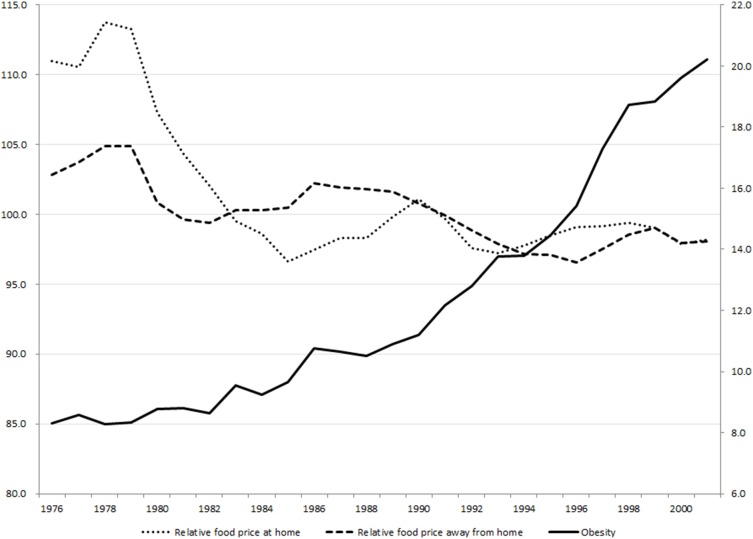
Relative Food Prices and the Obesity Prevalence in Metropolitan Areas: 1976–2001. Source: Authors’ calculation using consumer price indexes from the U.S. Bureau of Labor Statistics and NHIS respondents living in metropolitan areas.

Data used for the study came from a variety of databases, including the NHIS, the Consumer Price Index (CPI), the Current Population Survey (CPS), the County Business Patterns (CBP) and Historical Metropolitan Area Definitions (HMAD) for the time period of 1976 to 2001. Table 1 presents the summary statistics of the final sample and Fig. 1 illustrates changes in relative food prices and obesity prevalence during the time periods.

**Table 1:** Summary Statistics of Aggregate Samples: Weighted.

	Mean	Std. Dev.
**Dependent Variables**		
Δ Body Mass Index (BMI)	0.392	4.430
Δ Obesity	5.003	65.627
**Independent Variables at MSA Level**		
Δ Relative Food Prices at Home	−0.499	2.156
Δ Relative Food Prices away from Home	−0.333	1.884
Δ Female Hours of Work per Week	1.285	4.383
Δ Female Wage Rates	1.852	11.881
Δ Male Hours of Work per Week	−0.024	8.427
Δ Male Wage Rates	−0.016	2.702
Δ Unemployment Rates	−1.140	30.020
Δ Income per Capita	0.977	4.257
Δ Grocery/convenience stores (establishments per capita)	−0.374	10.188
Δ Restaurants (establishments per capita)	1.902	11.601
Δ Fitness & Recreational centers (establishments per capita)	2.629	17.464
**Independent Variables at Individual Level**		
Δ Family Income indicator 1 (≤ $14,999)	−6.328	60.003
Δ Family Income indicator 2 ($15,000∼$24,999)	−3.663	56.899
Δ Family Income indicator 3 ($25,000+)	5.707	40.977
Δ Family Income indicator 3 (missing)	5.249	63.844
Δ Age indicator 1 (18∼30)	−1.329	39.368
Δ Age indicator 2 (31∼45)	0.967	37.577
Δ Age indicator 3 (46∼60)	0.747	47.261
Δ Age indicator 4 (60+)	3.014	56.140
Δ Single	1.471	45.913
Δ Married	−0.806	26.654
Δ Others Marital Status	1.961	55.890
White	0.692	0.462
African American	0.141	0.348
Hispanic	0.130	0.336
Other Races	0.038	0.191
Less than High School	0.222	0.416
High School Graduates	0.353	0.478
Some College Education	0.202	0.402
College Graduates and above	0.222	0.416
Male	0.460	0.498
N	15,035	

#### Relative food prices

The relative food prices are the ratios of food price indexes to the price index for all items [9], [16]. The price indexes from 25 large metropolitan areas were identified in the CPI published by U.S. Bureau of Labor Statistics. Among them, Honolulu was excluded since the 1976–2001 waves of NHIS contained only 256 respondents from this metropolitan area. Phoenix was excluded because the food price indexes of this metropolitan area were available for only 5 years. Consequently, 23 large metropolitan areas included for the analysis were: Atlanta, Boston, Chicago, Cleveland, Dallas (including Fort Worth), Denver (including Boulder), Detroit (including Ann Arbor), Houston, Kansas City, Los Angeles (including Riverside), Miami (including Fort Lauderdale), Milwaukee, Minneapolis and St. Paul, New York, Philadelphia, Pittsburgh, Portland, San Diego, San Francisco (including Oakland), Seattle, St. Louis, Tampa (including St. Petersburg), and Washington (including Baltimore).

However, as pointed out by U.S. Bureau of Labor Statistics (www.bls.gov/cpi/cpiadd.htm), the CPI-Food price indexes are location specific and thus “the composition of the market basket and relative prices of goods and services in the market basket during the expenditure base period varies substantially across areas.” Therefore, the CPI-food price indexes of these large metropolitans are not directly comparable across these areas, although they are comparable over time within the same area. As a result, performing simple cross sectional analysis using any CPI figures directly would be inaccurate.

One way to get around this data issue is to compare the measures in *lo*g units. Therefore, we took log-log transformation in the fixed effect approach. Alternatively, we can compare the percentage changes in these price indexes across these metropolitan areas for the time period in the first-difference approach. Therefore, we constructed the percentage change of two relative food price variables using the equation (3),
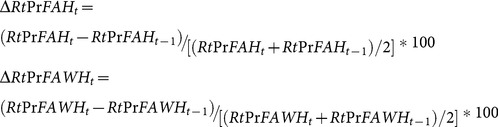
(3)


#### Body Weight and Demographic Variables

The measures of body weight and demographic characteristics came from the 1976–2001 waves of NHIS. The NHIS conducted by National Center for Health Statistics (NCHS), is designed to be the major source of information on the health of the civilian non-institutionalized population of the U.S. Since the smallest geographic identifiers available in the NHIS public use data are large metropolitan statistical areas (MSAs) and previous literature suggests that urbanization may affect body weight, the final samples used for the empirical analysis were limited to those from these MSAs. Because MSA identifiers have been excluded from the public used NHIS data since 2002, the data used for this analysis is censored in 2001.

Without missing values, the total sample size would be 18,400 (23 MSAs * 25 years * 4 race/ethnicity categories * 4 education categories * 2 gender categories). However, the food price indexes are not available for Miami before 1978, Tampa before 1987, and they are not available for Cincinnati for years 1988 and 1989, and Denver for 1988. As a result, the final panel contained 15,035 aggregate samples, representing 630,541 individual respondents (more than 99% of the total) living in these 23 large metropolitan areas.

We included two measures of body weight, body mass index (BMI) and a dichotomous indicator of obesity. BMI is calculated as 
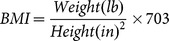
 and a respondent was categorized as obese if his or her BMI was greater than or equal to 30. Other demographic variables used in this study included gender, race/ethnicity, education attainment, marital status, age and family income. Except gender, race/ethnicity, and education, we obtained percentage changes in body weight measures and demographic variables for the first-difference estimation using equation (3).

#### Economic and population measures at the metropolitan level

Economic and population variables at metropolitan level included average wage rates and hours of work per week of both males and females, the average income per capita, unemployment rate, and the population size. They were obtained from the CPS March file and all nominal monetary terms were adjusted to 1982–1984 U.S. Dollars using the CPI.

#### Outlet density measures at metropolitan level

The density measures came from the CBP reports released by the U.S. Census Bureau. The purpose of CBP is to provide annual detailed geographic, industry, and other information for U.S. business establishments. For the purpose of this study, we aggregated the county-level information to the 23 large metropolitan areas. Because metropolitan areas are defined by the U.S. Office of Management and Budget and definitions change approximately every ten years based on Census data, counties included in a given metropolitan area may not be the same over the time. Consequently, we created a crosswalk between counties and metropolitan areas based on the HMAD to generate consistent geographic definitions between the NHIS and the CBP, and to minimize measurement errors in the density variables.

Specifically, we included the establishment information of five industries, according to the 6-digit code from the 1997 North American Industry Classification System (NAICS). These five industries are full-service restaurants (NAICS code: 722110), limited-service restaurants (722211), supermarkets and other grocery (except convenience) stores (445110), convenience stores (445120) and fitness and recreational sports centers (713940). Due to the consideration of the simplicity and the main purpose of this study, we collapsed the establishments of full-service restaurants with that of limited-service restaurants, and the establishments of supermarkets and other grocery with that of convenience stores. Using the establishment information from the CBP and the metropolitan population from the CPS, we finally constructed three metropolitan outlet density measures, the number of grocery and convenience stores per capita, the number of restaurants per capita, and the number of physical activity facilities per capita.

## Results

In this section, we first present our primary findings based on the first-difference approach. Then, we explore the potential heterogeneity of the impacts of relative food prices on body weight by education. Finally, we evaluate the robustness of our primary findings by using the fixed effect approach and by replicating the first-difference analysis with different aggregate samples obtained based on other criteria.

### Primary Results

Estimated relative food price effects on BMI and obesity are reported in Tables 2 and 3, respectively. The first two columns of these tables present the estimated effects of relative food prices on body weight measures, while the last two columns shows similar estimates after relaxing the linear restriction on time-varying variables. To investigate the robustness of these estimates, we excluded and included the outlet density measures of grocery and convenience stores, restaurants, and fitness & recreational centers in different specifications. In general, we found similar results with or without these density measures. It indicates that our first-difference estimates are plausibly exogenous to the influence of other omitted time-varying variables.

**Table 2:** The Estimated Effects of Relative Food Price Changes on BMI.

	Δ BMI^a,b^			
COEFFICIENT	(1)	(2)	(3)	(4)
Δ Relative Food Prices at Home	−0.02	−0.02	−0.02	−0.02
	(0.03)	(0.03)	(0.03)	(0.03)
Δ Relative Food Prices at Home Squared	-	-	0.00	0.00
	-	-	(0.00)	(0.00)
Δ Relative Food Prices away from Home	−0.01	−0.01	−0.02	−0.02
	(0.02)	(0.02)	(0.02)	(0.02)
Δ Relative Food Prices away from Home Squared	-	-	−0.01	−0.01
	-	-	(0.00)	(0.00)
Δ Grocery/convenience stores	-	−0.00	-	−0.01
	-	(0.01)	-	(0.01)
Δ Grocery/convenience stores squared	-	-	-	−0.00
	-	-	-	(0.00)
Δ Restaurants	-	0.01	-	0.01
	-	(0.01)	-	(0.01)
Δ Restaurants Squared	-	-	-	0.00
	-	-	-	(0.00)
Δ Fitness & Recreational centers	-	−0.00	-	0.00
	-	(0.01)	-	(0.01)
Δ Fitness & Recreational centers squared	-	-	-	−0.00
	-	-	-	(0.00)
Δ Metro Economic variables	Yes	Yes	Yes	Yes
Δ Metro Economic variables squared	No	No	Yes	Yes
n	15,035			

Notes: ^a^: All models are estimated using the first difference approach and based on weighted aggregate samples from the NHIS for years 1976 to 2001. ^b^: All regressions control for changes in demographic variables, including marital status, age and family income, as well as year fixed effects. Robust standard errors clustered at the metropolitan level in parentheses. ***p<0.01, **p<0.05, *p<0.1.

**Table 3:** The Estimated Effects of Relative Food Price Changes on Obesity.

	Δ Obesity^a,b^			
COEFFICIENT	(1)	(2)	(3)	(4)
Δ Relative Food Prices at Home	−1.07*	−0.93*	−1.35**	−1.18*
	(0.53)	(0.53)	(0.62)	(0.64)
Δ Relative Food Prices at Home Squared	-	-	−0.17	−0.16
	-	-	(0.11)	(0.11)
Δ Relative Food Prices away from Home	−0.51	−0.55	−0.73*	−0.82*
	(0.34)	(0.37)	(0.39)	(0.44)
Δ Relative Food Prices away from Home Squared	-	-	−0.02	−0.03
	-	-	(0.11)	(0.11)
Δ Grocery/convenience stores	-	−0.05	-	−0.17
	-	(0.27)	-	(0.28)
Δ Grocery/convenience stores squared	-	-	-	−0.01
	-	-	-	(0.01)
Δ Restaurants	-	0.37*	-	0.47**
	-	(0.20)	-	(0.17)
Δ Restaurants Squared	-	-	-	0.01*
	-	-	-	(0.00)
Δ Fitness & Recreational centers	-	−0.18	-	−0.17
	-	(0.18)	-	(0.17)
Δ Fitness & Recreational centers squared	-	-	-	−0.00
	-	-	-	(0.00)
Δ Metro Economic variables	Yes	Yes	Yes	Yes
Δ Metro Economic variables squared	No	No	Yes	Yes
n	15,035			

Notes: ^a^: All models are estimated using the first difference approach and based on weighted aggregate samples from the NHIS for years 1976 to 2001. ^b^: All regressions control for changes in demographic variables, including marital status, age and family income, as well as year fixed effects. Robust standard errors clustered at the metropolitan level in parentheses. ***p<0.01, **p<0.05, *p<0.

As presented in Table 2, we did not find any evidence that either the relative price of food at home or the relative price of food away from home had a significant influence on BMI. Similarly, we also did not find any evidence of associations between the density measures and BMI.

In Table 3, we found consistent evidence suggesting that the declines in relative prices of food at home were associated with increases in adult obesity. We also found some evidence showing the adverse association between relative prices of food away from home and obesity. Specifically, the results in the top panel of column 4 suggest that a 2.2% (one standard deviation) reduction in the relative prices of food at home was associated with a 3.3% increase in obesity and a 1.9% (one standard deviation) reduction in the relative prices of food away home was associated with a 1.7% increase in obesity.

The estimates in the bottom panel of columns 2 and 4 suggest that the number of restaurants per capita was also positively associated with increases in obesity among the adult population. Specifically, the results from the full specification (column 4) indicate that that an 11.6% (one standard deviation) increase in the number of restaurants per capita was associated with a 6.8% increase in obesity.

### Heterogeneous Impacts of Relative Food Prices by Education

Table 4 and Table 5 present estimated effects of relative food prices on body weight of the high-educated and the low-educated, respectively. The high-educated were defined as those with bachelor degrees or higher, while the low-educated contained those with high school diplomas only or less.

**Table 4:** The Estimated Effects of Relative Food Price Changes on BMI and Obesity: The High-Educated.

	Δ BMI^a,b^		Δ Obesity^a,b^	
COEFFICIENT	(1)	(2)	(3)	(4)
Δ Relative Food Prices at Home	0.02	0.03	−1.06	−1.06
	(0.06)	(0.06)	(1.46)	(1.89)
Δ Relative Food Prices at Home Squared	-	0.03***	-	0.25
	-	(0.01)	-	(0.45)
Δ Relative Food Prices away from Home	−0.05	−0.05	−0.31	−0.11
	(0.04)	(0.04)	(0.82)	(1.00)
Δ Relative Food Prices away from Home Squared	-	−0.03***	-	−0.11
	-	(0.01)	-	(0.31)
Δ Grocery/convenience stores	0.01	−0.00	0.04	−0.24
	(0.02)	(0.02)	(0.53)	(0.65)
Δ Grocery/convenience stores squared	-	−0.00	-	−0.02
	-	(0.00)	-	(0.01)
Δ Restaurants	0.02	0.03	0.50	0.78*
	(0.02)	(0.02)	(0.36)	(0.40)
Δ Restaurants Squared	-	0.00	-	0.01
	-	(0.00)	-	(0.01)
Δ Fitness & Recreational centers	−0.04***	−0.04***	−0.33	−0.27
	(0.01)	(0.01)	(0.37)	(0.37)
Δ Fitness & Recreational centers squared	-	−0.00	-	−0.00
	-	(0.00)	-	(0.00)
Δ Metro Economic variables	Yes	Yes	Yes	Yes
Δ Metro Economic variables squared	No	Yes	No	Yes
n	3,764			

Notes: ^a^: All models are estimated using the first difference approach and based on weighted aggregate samples from the NHIS for years 1976 to 2001. ^b^: All regressions control for changes in demographic variables, including marital status, age and family income, as well as year fixed effects. Robust standard errors clustered at the metropolitan level in parentheses. ***p<0.01, **p<0.05, *p<0.1.

**Table 5:** The Estimated Effects of Relative Food Price Changes on BMI and Obesity: The Low-Educated.

	Δ BMI^a,b^		Δ Obesity^a,b^	
COEFFICIENT	(1)	(2)	(3)	(4)
Δ Relative Food Prices at Home	−0.04	−0.04	−1.49***	−1.57***
	(0.04)	(0.04)	(0.49)	(0.48)
Δ Relative Food Prices at Home Squared	-	−0.00	-	−0.18*
	-	(0.00)	-	(0.10)
Δ Relative Food Prices away from Home	−0.01	−0.03	−0.43	−0.84*
	(0.03)	(0.03)	(0.43)	(0.42)
Δ Relative Food Prices away from Home Squared	-	0.00	-	0.03
	-	(0.01)	-	(0.13)
Δ Grocery/convenience stores	0.00	−0.01	0.23	0.11
	(0.02)	(0.02)	(0.39)	(0.36)
Δ Grocery/convenience stores squared	-	−0.00	-	0.00
	-	(0.00)	-	(0.01)
Δ Restaurants	0.01	0.01	0.08	0.21
	(0.02)	(0.02)	(0.30)	(0.27)
Δ Restaurants Squared	-	0.00	-	0.00
	-	(0.00)	-	(0.00)
Δ Fitness & Recreational centers	0.00	0.00	−0.19	−0.20
	(0.01)	(0.01)	(0.19)	(0.18)
Δ Fitness & Recreational centers squared	-	−0.00	-	−0.00
	-	(0.00)	-	(0.00)
Δ Metro Economic variables	Yes	Yes	Yes	Yes
Δ Metro Economic variables squared	No	Yes	No	Yes
n	7,604			

Notes: ^a^: All models are estimated using the first difference approach and based on weighted aggregate samples from the NHIS for years 1976 to 2001. ^b^: All regressions control for changes in demographic variables, including marital status, age and family income, as well as year fixed effects. Robust standard errors clustered at the metropolitan level in parentheses. ***p<0.01, **p<0.05, *p<0.1.

The first two columns of Table 4 present the impacts of relative food prices on BMI of the high-educated, while the latter two columns indicate such impacts on obesity. These estimates suggest that the relative food prices had little impact on the BMI of the high-educated. The results on the relative prices of food at home in the last two columns were similar to the findings from the general population in terms of the magnitude but insignificant. The results on the relative prices of food away from home for the high-educated were both smaller and insignificant, compared with estimates from the general population.

We found consistent evidence of the inverse relationship between the number of fitness and recreational centers per capita and BMI of the high-educated, although the estimated impact was fairly small. However, we did not find similar evidence on obesity. In sum, the results in column 2 suggest that a 17.5% (one standard deviation) increase in the number of fitness and recreational centers per capita was associated with a 0.7% reduction in BMI among the high-educated.

We also found a stronger connection between the number of restaurants per capita and obesity among the high-educated, compared with that among the general population. The phenomenon might be correlated with higher income associated with higher education. Consequently, they were more likely to afford meals outside house. Specifically, our estimates in column 4 suggest that an 11.6% (one standard deviation) increase in the number of restaurants per capita was associated with a 10.4% growth in obesity among the high-educated.

Table 5 presents the estimated impacts of relative food prices among the low-educated. Likewise, we did not find any evidence indicating that relative food prices had substantial impacts on BMI. We found little impact of the metropolitan outlet density measures on either measures of body weight. This might be related to the lack of health knowledge among the low-educated. Alternatively, this phenomenon may be related to lower income among this population. As a result, they might have limited access to healthy foods, full-service restaurants, or the gym membership because of financial constrain.

We found consistent evidence showing a larger and stronger inverse association between relative food prices and obesity among this disadvantaged population. The estimates suggest that a 2.2% (one standard deviation) reduction in the relative prices of food at home was associated with a 4.2% increase in the obesity prevalence and a 1.9% (one standard deviation) reduction in the relative prices of food away home was associated with a 1.5% increase in obesity.

In sum, we did not find evidence for the influence of relative food prices on BMI among the high- or low- educated population, but we found consistent evidence of such impacts on obesity. Interestingly, our findings indicate that the effects of relative food prices on obesity were much more pronounced among the low-educated. In contrast, the effects of relative food prices were somewhat muted among the high-educated, although both the signs and the magnitude of these estimates remained comparable to those from the general population.

### Sensitivity Analyses

In addition to the first-difference analysis, the pseudo-panel also enables us to investigate the effect of relative food prices using the fixed effect approach. By taking the log transformation on both sides of the equation, the estimators of relative food price at home and relative food price away from home can also be interpreted as price elasticities of demand. Table 6 presents these estimates.

**Table 6:** The Fixed Effect Estimates of Relative Food Price on Body Weight Measures.

	Body Weight Measures^a,b^					
	Full Sample		The High-Educated		The Low-Educated	
COEFFICIENTS	(1)	(2)	(3)	(4)	(5)	(6)
	Log BMI	Log Obesity	Log BMI	Log Obesity	Log BMI	Log Obesity
Log Relative Food Prices at Home	−0.03	−1.19*	−0.00	−0.45	−0.05	−1.48**
	(0.02)	(0.63)	(0.03)	(0.92)	(0.03)	(0.63)
Log Relative Food Prices away from Home	−0.01	0.18	−0.00	−0.53	−0.00	0.29
	(0.01)	(0.46)	(0.02)	(0.58)	(0.01)	(0.44)
Log Grocery/convenience stores	−0.01	−0.53**	−0.02**	−0.69***	−0.01	−0.19
	(0.01)	(0.20)	(0.01)	(0.24)	(0.01)	(0.22)
Log Restaurants	0.00	0.15	0.00	−0.12	0.00	−0.01
	(0.01)	(0.20)	(0.01)	(0.40)	(0.01)	(0.23)
Log Fitness & Recreational centers	0.01	0.26	0.00	0.44	0.01	0.09
	(0.01)	(0.20)	(0.01)	(0.30)	(0.01)	(0.24)
Metro Economic variables	Yes	Yes	Yes	Yes	Yes	Yes
N	15,289		3,830		7,724	

Notes: ^a^: All models are estimated using the fixed effect approach and based on weighted aggregate samples from the NHIS for years 1976 to 2001. ^b^: All regressions control for demographic variables, including marital status, age and family income, indicators of gender, race/ethnicity, and education, as well as year and metropolitan fixed effects. Robust standard errors clustered at the metropolitan level in parentheses. ***p<0.01, **p<0.05, *p<0.1.

To be consistent, we first obtained the estimated relative price effects using the full sample and then explored the heterogeneity by education. The estimated relative price effects on BMI are presented in columns 1, 3, and 5, while such impacts on obesity are presented in columns 2, 4, and 6. The estimates in Table 6 are generally consistent with the findings from the first-difference approach. We failed to find any association between relative food prices and BMI, but we found a significant association between relative food prices at home and obesity. Particularly, the estimated elasticities of relative food prices at home presented in Table 6 are comparable to those estimates from the first-difference model: the impact was more pronounced for the low-educated and turned insignificant for the high-educated. We did not find a significant association between relative food prices away from home and obesity. This is not a surprising finding, since the effects found in the first-difference approach are only marginally significant and depend on the specification.

Sensitivity estimates from the two alternative pseudo-panels based on different aggregate samples are reported in Tables 7 and 8. Table 7 presents the estimated effects of the relative food prices on BMI, while Table 8 reports such impacts on obesity.

**Table 7:** The Estimated Effects of Relative Food Price Changes on Changes in Body Mass Index: By Grouping Method.

	Δ BMI^a,b^					
Aggregating Methods	Gender + Race + Edu		Gender + Race		Gender only	
COEFFICIENTS	(1)	(2)	(3)	(4)	(5)	(6)
Δ Relative Food Prices at Home (percentage)	−0.02	−0.02	−0.02	−0.02	−0.01	−0.02
	(0.03)	(0.03)	(0.03)	(0.03)	(0.03)	(0.03)
Δ Relative Food Prices at Home Squared (percentage)	-	0.00	-	0.00	-	0.00
	-	(0.00)	-	(0.00)	-	(0.00)
Δ Relative Food Prices away from Home (percentage)	−0.01	−0.02	−0.01	−0.02	−0.02	−0.02
	(0.02)	(0.02)	(0.02)	(0.02)	(0.02)	(0.02)
Δ Relative Food Prices away from Home Squared (percentage)	-	−0.01	-	−0.01*	-	−0.01*
	-	(0.00)	-	(0.00)	-	(0.00)
Δ Grocery/convenience stores	−0.00	−0.01	−0.00	−0.01	0.00	−0.01
	(0.01)	(0.01)	(0.01)	(0.01)	(0.01)	(0.01)
Δ Grocery/convenience stores squared	-	0.00	-	−0.00	-	−0.00
	-	(0.01)	-	(0.00)	-	(0.00)
Δ Restaurants	0.01	0.01	0.01	0.01	0.01	0.01
	(0.01)	(0.01)	(0.01)	(0.02)	(0.01)	(0.02)
Δ Restaurants Squared	-	0.00	-	0.00	-	0.00
	-	(0.01)	-	(0.00)	-	(0.00)
Δ Fitness & Recreational centers	0.00	0.00	−0.00	−0.00	−0.00	−0.00
	(0.01)	(0.01)	(0.01)	(0.01)	(0.01)	(0.01)
Δ Fitness & Recreational centers squared	-	−0.00	-	−0.00	-	−0.00
	-	(0.00)	-	(0.00)	-	(0.00)
Δ Metro Economic variables	Yes	Yes	Yes	Yes	Yes	Yes
Δ Metro Economic variables squared	No	Yes	No	Yes	No	Yes
N	15,035		4,296		1,112	

Notes: Notes: ^a^: All models are estimated using the first difference approach and based on weighted aggregate samples from the NHIS for years 1976 to 2001. ^b^: All regressions control for changes in demographic variables, including marital status, age and family income, as well as year fixed effects. Robust standard errors clustered at the metropolitan level in parentheses. ***p<0.01, **p<0.05, *p<0.1.

**Table 8:** The Estimated Effects of Relative Food Price Changes on Changes in Obesity: By Grouping Method.

	Δ Obesity^a,b^					
Aggregating Methods	Gender + Race + Edu		Gender + Race		Gender only	
COEFFICIENTS	(1)	(2)	(3)	(4)	(5)	(6)
Δ Relative Food Prices at Home (percentage)	−0.93*	−1.18*	−1.01**	−1.25**	−0.86*	−1.04*
	(0.53)	(0.64)	(0.48)	(0.55)	(0.44)	(0.53)
Δ Relative Food Prices at Home Squared (percentage)	-	−0.16	-	−0.15*	-	−0.14
	-	(0.11)		(0.08)	-	(0.11)
Δ Relative Food Prices away from Home (percentage)	−0.55	−0.82*	−0.45	−0.72*	−0.33	−0.62
	(0.37)	(0.44)	(0.36)	(0.40)	(0.35)	(0.40)
Δ Relative Food Prices away from Home Squared (percentage)	-	−0.03	-	−0.02	-	−0.01
	-	(0.11)		(0.10)	-	(0.08)
Δ Grocery/convenience stores	−0.05	−0.17	−0.03	−0.14	−0.08	−0.22
	(0.27)	(0.28)	(0.26)	(0.27)	(0.26)	(0.29)
Δ Grocery/convenience stores squared	-	−0.01	-	0.00	-	−0.00
	-	(0.01)	-	(0.00)	-	(0.00)
Δ Restaurants	0.37*	0.47**	0.39	0.48**	0.47	0.57*
	(0.20)	(0.17)	(0.24)	(0.22)	(0.31)	(0.29)
Δ Restaurants Squared	-	0.01*	-	0.01	-	0.01*
	-	(0.00)	-	(0.00)	-	(0.00)
Δ Fitness & Recreational centers	−0.18	−0.17	−0.19	−0.18	−0.24	−0.25
	(0.18)	(0.17)	(0.17)	(0.16)	(0.16)	(0.15)
Δ Fitness & Recreational centers squared	-	−0.00	-	−0.00*	-	−0.00
	-	(0.00)	-	(0.00)	-	(0.00)
Δ Metro Economic variables	Yes	Yes	Yes	Yes	Yes	Yes
Δ Metro Economic variables squared	No	Yes	No	Yes	No	Yes
N	15,035		4,296		1,112	

Notes: ^a^: All models are estimated using the first difference approach and based on weighted aggregate samples from the NHIS for years 1976 to 2001. ^b^: All regressions control for changes in demographic variables, including marital status, age and family income, as well as year fixed effects. Robust standard errors clustered at the metropolitan level in parentheses. ***p<0.01, **p<0.05, *p<0.1.

For the comparison purpose, the first two columns in Table 7 simply duplicate our primary estimates, which are from columns 2 and 4 of Table 2. The results shown in columns 3 and 4 are base on the first alternative scenario, in which only two demographic variables were used to aggregate NHIS respondents. Thus, the total sample size was smaller and should be 4,600 if there were no missing information. But because of the missing values for metropolitan food price indexes in certain years, the final sample used for this sensitivity analysis was 4,296. Similarly, the alternative aggregate samples used to produce the estimates in columns 5 and 6 were constructed based on the second alternative scenario, in which gender was the only demographic criterion. Hence, the final sample size should be 1,150 without missing values and it was 1,112 because of the missing information.

By comparing the estimates across the columns in Tables 7 and 8, it is clear that the findings are consistent across different aggregation methods, in term of both signs and magnitude. These estimates provide strong supporting evidence for using aggregate samples.

## Discussion

We extend the existing literature in this area by focusing on relative food prices. We examined the impacts of relative prices of food at home and food away from home on BMI and obesity, by using pseudo-panel data created from NHIS individual respondents living in large metropolitan areas for the time period between 1976 and 2001. Based on the pseudo-panel data, we used both first-difference and fixed effect approaches to provide estimates that could be possibly interpreted as causal.

We also include a wide variety of controls for local time-varying factors and female labor participation, including unemployment rate, average income per capita, male and female wage rates and hours of work per week from 1976 to 2001 to strengthen the causal interpretation. It is impossible to rule out all possible sources of local heterogeneity that might create biases in our estimated impacts of relative food prices on BMI and obesity. However, by excluding and including the metropolitan outlet density measures of grocery stores, restaurants and fitness and recreational centers in our empirical analysis, we fail to find evidence of such impact from these most likely sources. We also note that the different empirical approaches, specifications, and alternative aggregate samples produced very consistent estimates of the association between relative food prices and obesity.

We find strong evidence that relative price of food at home were inversely associated with obesity. Such associations of relative price of food at home were much more pronounced among the low-educated and somewhat muted among the high-educated. Our conclusions are largely consistent with the existing evidence, suggesting that both child and adult body weight in low- socioeconomic families was more sensitive to absolute food prices than that of their counterparts from non-poor households [37], [39], [46].

We did not find consistent evidence that relative food prices have a significant impact on BMI. These estimates indicate that the changes in relative food prices may have disproportional large impacts on those who are at the risk for obesity. This result is also mirror findings from existing literature [8], [35]. For example, estimates from Quantile regressions indicate that the price of energy-dense foods had larger impact on body weight of adolescents in the top quintile of the conditional distribution of BMI [35].

According to U.S. Department of Labor, the relative price of food at home was 1.11 and that of food away from home was 1.02 in 1976, while the relative prices of food at home and food away from home were both 0.98 in 2001. Consequently, the relative prices of food at home and food away from home declined 12.5% and 4.1% during this time period, respectively. Our findings imply that the reduction in the relative prices of food at home from 1976 to 2001 contributed to a 14.7% growth in obesity prevalence and that the reduction in the relative prices of food away from home might add another 3.4% increase in obesity prevalence among adults living in metropolitan areas. That said, approximately 18.1% of growth in obesity from 1976 to 2001 could be attributed to the changes in relative food prices.

It is important to note that these conclusions should be interpreted with caution. Our findings are limited to U.S. large metropolitan areas during the analytical time period that is between 1976 and 2001. Therefore, more research based on a broader population and/or more recent data is certainly needed. Since our samples are limited to large metropolitan areas, that might be the reason that we found little association between the density measures and body weight. Also, self-reported body weight used in the analysis may be subject to measurement errors.

Comparing with the existing literature, we find modest effects of relative food prices on obesity. From a policy perspective, these results suggest that policies raising food prices, like “fat taxes” or other pricing disincentives for energy intake will have a moderate impact on obesity in the short term. These policies are likely to have their greatest impact on lower income and younger populations whose consumption decisions are more sensitive to price. The better tax base - high-fat, energy dense foods or carbohydrate-rich sugar sweetened foods – also remains to be better understood.
